# A Handheld Laser-Scanning-Based Methodology for Monitoring Tree Growth in Chestnut Orchards

**DOI:** 10.3390/s24061717

**Published:** 2024-03-07

**Authors:** Dimas Pereira-Obaya, Carlos Cabo, Celestino Ordóñez, José Ramón Rodríguez-Pérez

**Affiliations:** 1 Grupo de Investigación en Geomática e Ingeniería Cartográfica (GEOINCA), Universidad de León, Avenida de Astorga sn, 24401 Ponferrada, Spain; jr.rodriguez@unileon.es; 2 Department of Mining Exploitation and Prospecting, Escuela Politécnica de Mieres, Universidad de Oviedo, 33600 Mieres, Spain; carloscabo@uniovi.es (C.C.); ordonezcelestino@uniovi.es (C.O.)

**Keywords:** sweet chestnut, MLS, SLAM, 3-D point cloud, tree growth monitoring

## Abstract

Chestnut and chestnut byproducts are of worldwide interest, so there is a constant need to develop faster and more accurate monitoring techniques. Recent advances in simultaneous localization and mapping (SLAM) algorithms and user accessibility have led to increased use of handheld mobile laser scanning (HHLS) in precision agriculture. We propose a tree growth monitoring methodology, based on HHLS point cloud processing, that calculates the length of branches through spatial discretization of the point cloud for each tree. The methodology was tested by comparing two point clouds collected almost simultaneously for each of a set of sweet chestnut trees. The results obtained indicated that our HHLS method was reliable and accurate in efficiently monitoring sweet chestnut tree growth. The same methodology was used to calculate the growth of the same set of trees over 37 weeks (from spring to winter). Differences in week 0 and week 37 scans showed an approximate mean growth of 0.22 m, with a standard deviation of around 0.16 m reflecting heterogeneous tree growth.

## 1. Introduction

Sweet chestnut (*Castanea sativa* Mil.) coppices and orchards have been managed for their economic value by humans worldwide, and especially in Europe [1,2]. Both wood and nut products may be affected by abiotic and biotic problems; the most important threats to chestnut orchards in recent years include the Asian chestnut gall wasp (*Dryocosmus kuriphilus* Yasumatsu), ink disease (*Phytophthora cinnamomi*), and chestnut blight (*Cryphonectria parasitica*). Climate change also affects the proper development of chestnut trees, as it accelerates the expansion of pests and the emergence of other pathogens [3,4].

An interest in woody crops and the consolidation of remote sensing techniques as a non-destructive data collection method have contributed to the development of precision agriculture [5]. Active and passive remote sensors have been successfully used for vegetation monitoring, for purposes such as species classification, health status, and tree allometry assessment. Light detection and ranging (LiDAR) techniques have been demonstrated to be an efficient and accurate method for distinguishing between trees and vegetation in urban environments [6,7]. Spectral imaging and LiDAR data have been combined to identity woody crop species [8] in different kinds of orchards, including sweet chestnut. Pádua et al. [9] used spectral images captured by unmanned aerial vehicles (UAVs) to assess chestnut health status, while Rivera et al. [5] reviewed the use of LiDAR data for health monitoring in other woody crops. In the context of agriculture, lands as habitats for wildlife species, Sánchez-Díaz et al. [10] have used LiDAR to calculate cocoa plantation height.

The use of LiDAR data, especially from ground-based laser scanning platforms—terrestrial laser scanning (TLS), handheld laser scanning (HHLS), and mobile laser scanning (MLS)—has acquired significant prominence in forestry applications, particularly in the context of wood production monitoring [11,12]. This technology, by allowing for the precise and efficient measurement of forest resources, contributes to sustainable forest management practices. Ground-based laser scanning creates detailed and realistic three-dimensional representations of the forest environment, capturing accurate data on tree size, structure, and density. This information aids forestry professionals in the identification of optimal harvesting areas, tree growth patterns, and overall forest health. There is a large body of research that highlights the advantages of ground-based laser scanning in comparison to traditional field methods for forest inventory, mensuration, and monitoring. In providing high-resolution spatially explicit data, ground-based laser scanning is becoming established in precision forest inventory as a non-destructive but reliable means of characterizing crop structures and geometry. TLS, in particular, has been used by Schindler et al. [13] and Wang et al. [14] to analyze walnut tree allometry and fruit location, respectively, by Schindler et al. [15] to estimate tree parameters in a wild cherry agroforestry system, and by Torres-Sánchez et al. [16] to estimate tree crown parameters and characterize pear and peach orchards geometries.

Remote sensing techniques have been specifically tested in chestnut orchards using different scales and platforms. Alonso et al. [17] used satellite images and low-density LiDAR to identify trees and estimate their heights. UAV imagery has been used for health monitoring and nutritional deficiency identification and even to calculate biomass in non-regular chestnut orchards [9]. High-resolution UAV images have automatically identified chestnut fruits and estimated production and yield [18]. HHLS has also very recently been used by Balestra et al. [19] to model monumental chestnut trees. MLS, based on HHLS with an integrated simultaneous localization and mapping (SLAM) algorithm, offers exceptional flexibility and mobility, overcoming some problems associated with the use of multi-scan TLS methods. However, the downside of MLS compared to TLS lies in its limited accuracy and the presence of noise [20,21].

Some research describes methods for analyzing tree growth from TLS point clouds, focusing on specific parameters, such as stem diameters [22,23], where the full-potential geometric coherence and accuracy inherent to TLS point clouds are used. However, mainly due to the aforementioned lower accuracy and higher presence of noise, as far as we are aware, there is a research gap in the methods that use MLS point clouds for analyzing tree growth.

The main objective of this work is to provide an efficient and easy-to-implement HHLS-based methodology for monitoring individual tree growth in chestnut orchards, based on comparing point clouds from different epochs. Specifically, the proposed methodology simplifies the comparison by using distance images obtained after discretizing the point clouds.

## 2. Materials and Methods

### 2.1. Study Site and MLS Data Acquisition

**Figure 1:**
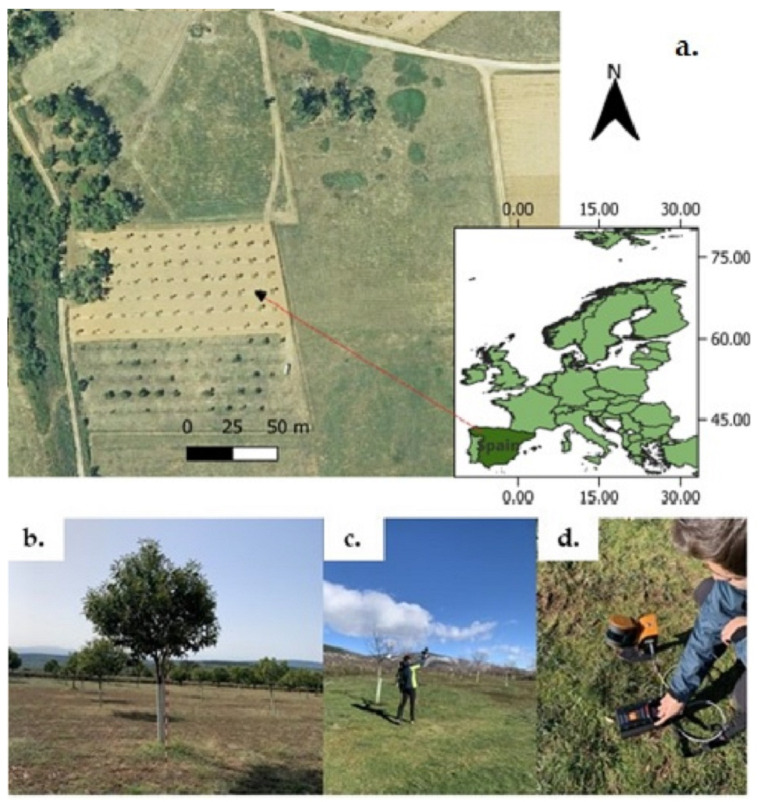
(**a**): Location map of the study area (42°42′27.65″ N, 6°26′13.54″ W; WGS84); (**b**): A chestnut tree in the plantation; (**c**,**d**): Data acquisition using the GeoSLAM ZEB Horizon.

The methodology was validated in a 1.4 ha sweet chestnut orchard located in northwestern Spain, specifically in Robledo de las Traviesas (El Bierzo, Castilla y León; Figure 1a). Due to infestation, a field campaign, including HHLS measurements, was implemented to control the health status of 64 trees of the same variety and age.

Data were collected with a GeoSLAM ZEB Horizon (GeoSLAM Ltd., Nottingham, UK; Figure 1c,d), which integrates a 16-laser-beam Velodyne Puck LITE sensor (Velodyne LiDAR Inc., San Jose, CA, USA). Its field of view, 360° × 270°, captures points at a rate of up to 300,000 points per second. Working over a range of 10 m, the scanner has an accuracy of 0.01–0.03 m. The horizontal and vertical angular resolutions are 0.2° and 2°, respectively. These technical characteristics, together with the speed and simplicity in data collection (the scanner weighs only 1.45 kg), as well as the size of the orchard, make this equipment ideal for this specific work compared to other systems such TLS or LiDAR drone. TLS systems provide more accurate measurements but at the cost of more expensive equipment and much slower data collection. For its part, LiDAR drone systems also require more expensive equipment, greater experience in data collection, and have limitations in obtaining points in the lateral and lower areas of the treetops.

The software suite provided with the scanner, GeoSLAM hub 6.2.1 (GeoSLAM Ltd., Nottingham, UK), uses a SLAM implementation that allows the generation of coherent point clouds in a common Cartesian coordinate system. In order to bring extra robustness, data collection trajectories were defined with the support of ground control points georeferenced in the field using the Global Navigation Satellite System (GNSS). In addition, to minimize the drift in the scans associated with SLAM uncertainties, the trajectories were designed taking into account their length, the duration of the measurement, and the presence of enough anchor features to create a coherent point cloud. 

To assess growth over a complete season (37 weeks), 3 HHLS scans were taken in leaf-off conditions: Scan 1 and Scan 2 just before the beginning of the growing season (6 May 2022), and Scan 3 once the trees had shed their leaves in winter (24 January 2023). Scans 1 and 2, taken the same day, were used to validate our methodology.

Using CloudCompare software 2.12.4 [24], the three point clouds were denoised and segmented to extract the trees, obtaining point clouds of individual trees. The average density of these point clouds was ~3200 points/m^2^.

### 2.2. Tree Point Cloud Processing

**Figure 2:**
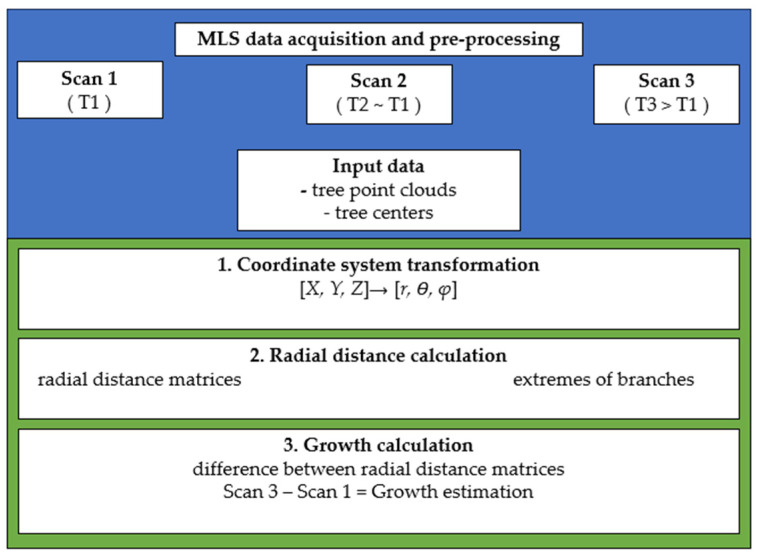
Tree growth monitoring workflow. HHLS data acquisition and pre-processing (upper blue section), where T1, T2, and T3 refer to the time of each scan, and growth monitoring steps (lower section): 1. Coordinate system transformation; 2. Radial distance calculation; and 3. Growth calculation.

Our method is based on a geometric conception of the studied trees, resembling a radial spherical structure. In this model, branch growth is simplified by being quantified longitudinally in the direction the branches diverge from a central point. This allows for a more streamlined analysis of tree growth, as it focuses on the primary growth vectors of the branches as they extend outward. By reducing the complex structure of the tree to a more manageable spherical model, we can more effectively measure and understand growth patterns, and particularly how the branches evolve over time in relation to the tree’s central mass. The methodology automatically identifies the branch ends, by calculating, for each tree, radial distances from a central point *C_i_* (*i* = 1, …, *n*), where *n* is the number of trees (Figure 2). Those central points correspond to the locations where the trunk begins to divide into branches. The method was implemented in Python code v 3.9.12 [25], with individual tree point clouds and their corresponding center points (*C_i_*) as inputs. 

As depicted in the flowchart in Figure 2, the procedure has 3 main steps:

1. Transformation of the tree point clouds from Cartesian to polar coordinates with an origin in the center of mass of each tree. As explained above, this transformation allows to leverage the radial structure of the branches to estimate their length.
2. Point cloud discretization in radial sections to calculate branch length. This discretization makes it easier to detect the ends of the branches.
3. Comparison of branch ends measured on two different dates to estimate tree growth over time. In essence, the comparison is carried out by subtracting matrices whose elements are distances to the central point.

#### 2.2.1. Radial Distance and Branch End Estimation

For a set of individual tree point clouds and centers (*C_i_*), the ends of the branches were located, and the distances to their corresponding center (*C_i_*) were calculated. Using Equations (1)–(3), the tree point cloud Cartesian coordinate system (*X*, *Y*, *Z*) was transformed into a spherical coordinate system (*r*, *ϴ*, *φ*) centered in *C_i_*. In the spherical coordinate system, the azimuthal angle (*ϴ*) and zenithal angle (*φ*) go from 0° to 360° and from 0° to 90°, respectively.

(1) $$r=\sqrt{X^{2}+Y^{2}+Z^{2}}$$

(2) $$\;\theta =\arctan\frac{X}{Y}$$

(3) $$\varphi =\arcsin\frac{Z}{r}$$

**Figure 3:**
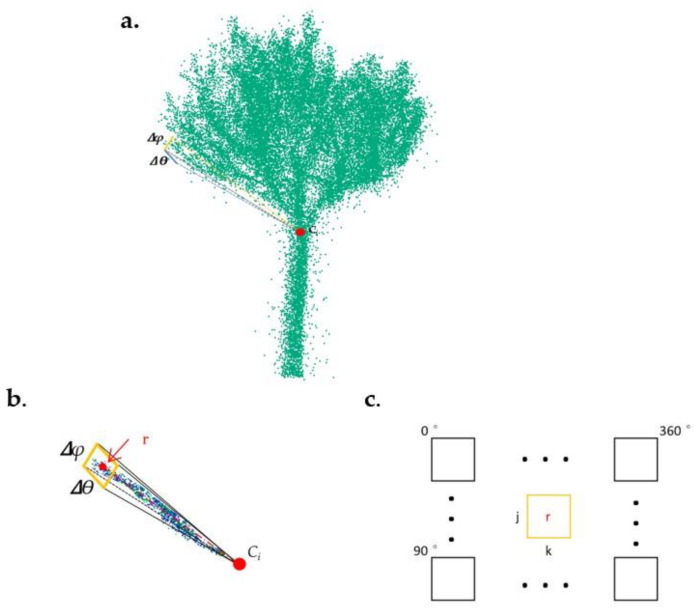
Branch ends detection and radial distance calculations. (**a**). A solid angle over a tree point cloud (the coordinate system center, *Ci*, is indicated by the red dot), (**b**). The denoised point cloud within the solid angle and its radial distance (broken red line), (**c**). The radial distance to the furthest point in cell [*j*, *k*] defined by the solid angle (Δ*ϴ*, Δ*φ*).

The point cloud of each tree was discretized in solid angles delimited by specific increments, Δ*θ* and Δ*φ*, of the azimuthal and zenithal angles, respectively (Figure 3a). Based on this discretization, a matrix was created with as many rows (*j*) and columns (*k*) as resulted from dividing 90° (*φ*) and 360° (*ϴ*) by their respective angular increments. After removing possible outliers based on distance percentiles, the radial distance from *C_i_* to the furthest point in each solid angle was calculated. Those values were stored in the position [*j*, *k*] in the matrix (Figure 3c).

#### 2.2.2. Growth Estimation

**Figure 4:**
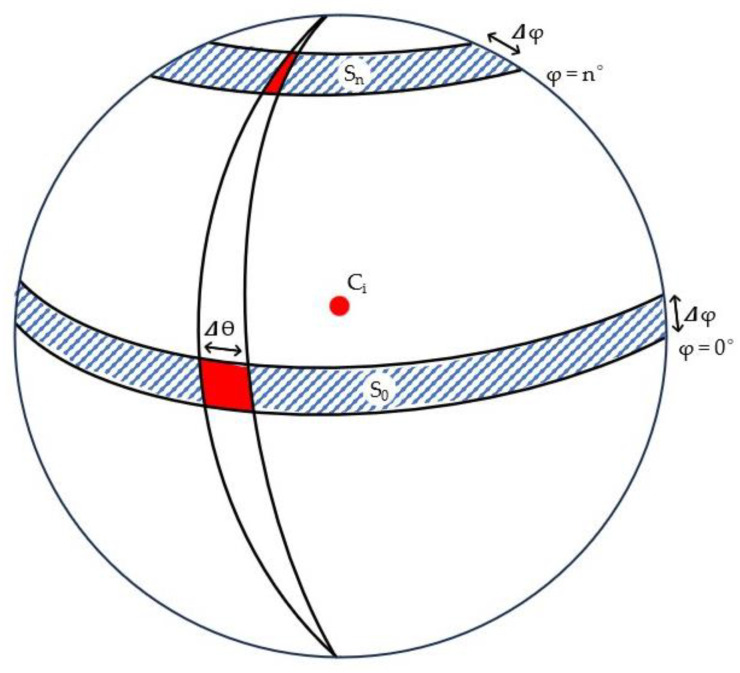
Representation of the spherical segments (blue), with the azimuthal angle *ϴ* and zenithal angle *φ* delimiting spherical quadrilaterals (red). The spherical segment surfaces *S*_0_ and *S_n_* depend on *φ*: the surface is greatest when φ is close to 0° and thus *S*_0_ *> S_n_*. Accordingly, the spherical quadrilaterals also have a surface decreasing with *φ*.

Angular increments in the azimuthal and zenithal angles (Δ*ϴ*, Δ*φ*) delimited spherical quadrilaterals in spheres of radius *r*, whose size decreased with an increasing zenithal angle (*φ*), as shown in Figure 4. Thereafter, the corresponding planar quadrilaterals defining the matrices storing the radial distances were reshaped so that they all covered the same area regardless of the zenithal angle. The reshaping was performed by joining quadrilaterals/cells (Figure 3 and Figure 4) in each spherical segment (*S_n_*), so the area of each quadrilateral is equivalent to that on the horizontal segment (*S*_0_), as in *S*_0_ $=\;S_{n}r\;\cos\varphi .$

**Figure 5:**
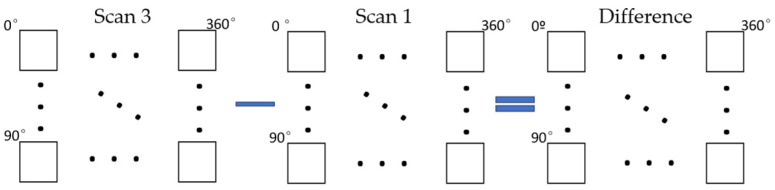
Procedure for calculating the growth of each tree. Growth is calculated as the average difference between the radial distance matrices corresponding to two different dates (e.g., Scan 3 and Scan 1). The squares, which represent elements of a matrix, store the maximum distances from the origin of the coordinates to the ends of the branches.

As shown in Figure 5, we assume that the growth of each tree can be approximated by the average difference between the radial distance matrices of Scan 1 and Scan 3.

## 3. Results and Discussion

### 3.1. Validation

In our study, as explained in Section 2.1, we employed a specific methodology that hinges on comparing two scans taken under identical conditions on the same day. This approach is crucial because it allows us to attribute any observed differences exclusively to the measurement system. By comparing two identical-condition scans, we aim to evaluate the precision of our measurement system. Ideally, in a scenario where the scans are perfectly aligned, the difference should be zero. Consequently, we expect our metrics—mean error (ME), mean absolute error (MAE), and standard deviation (SD) of the means—to closely approximate zero, indicating minimal to no differences between scans. 

Table 1 presents these metrics: ME represents the bias in our methodology, MAE quantifies the magnitude of distance errors without considering their direction, and SD (σ) measures the dispersion of tree growth variations within the orchard. These statistics collectively indicate that the method is unbiased, and that error is negligible, as it falls within the scanner’s accuracy range (0.01–0.03 m). Furthermore, the fact that the SD of the differences in radial distances is consistent across various resolutions confirms the reliability and precision of our measurement technique within the defined accuracy limits.

Regarding the effect of the resolution, it can be seen from Table 1 that there are hardly any differences in the estimates of mean tree growth, indicating no clear criterion for an optimal resolution value. However, lower resolutions (corresponding to quadrilaterals with shorter sides) would not be appropriate, and nor would higher resolutions be advisable, since there could be several branches in the same quadrilateral. Therefore, intermediate resolutions were considered to be the most appropriate. Thus, for resolutions of 3° or 5°, the side of the quadrilateral at a distance of 1.5 m (approximately the maximum branch length) would be about 8 cm and 13 cm, respectively, which would seem to be reasonable values to estimate branch growth.

### 3.2. Growth Analysis

The validation analysis confirmed that the proposed methodology was well suited for estimating tree growth over time, since the analysis of the differences between scans performed almost simultaneously and under identical conditions, measured by ME, MAE, and SD statistics, led to consistent growth values very close to zero, as expected. Then, we can state that our method is able to estimate differences between branches in two scans that are within the precision of the data and that there is not a significant bias due to systematic errors that could lead to erroneous estimates of tree growth.

**Figure 6:**
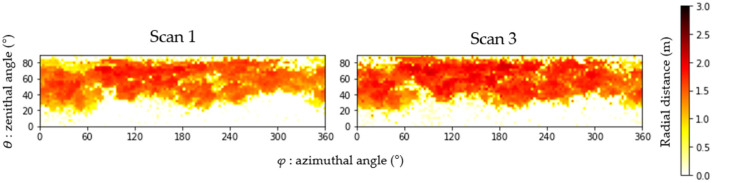
Radial distance matrices for Scan 1 and Scan 3 displayed as images. Radial distance is represented in meters, and azimuthal (*ϴ*) and zenithal (*φ*) angles in degrees.

Accordingly, growth from spring to winter (Scan 1 to Scan 3), for the 64 individual trees, was analyzed at different resolutions. Applying the automatic algorithm, the radial distance in each angular sector from tree center to the furthest point was calculated (represented as 18 rows and 72 columns, equal to 1296 cells/pixels), and branch ends were identified in the point clouds. Figure 6 shows the radial distances calculated for a resolution of 5° as an image composed of 1296 cells/pixels (18 × 72). As can be seen, for 0° ≤ *φ* ≥ 20°, it is difficult to find points in the cloud. Progressively darker red tones correspond to progressively larger branches, a distribution due to tree crown shape repeated in all the trees (see Figure 6). 

**Figure 7:**
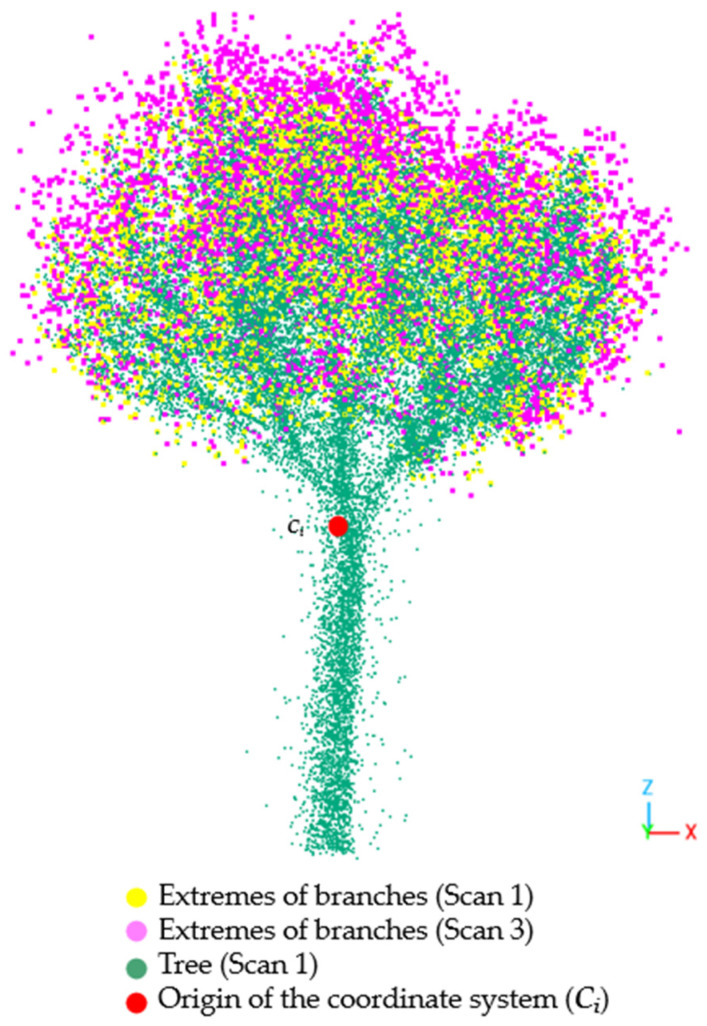
Scan 1 and Scan 3 branch ends represented on a tree point cloud (Scan 1 as reference). The red dot represents the coordinate system center (*C_i_*) from where the radial distances were calculated.

Figure 7, which depicts the branch extremes for Scan 1 (yellow) and Scan 3 (purple), clearly indicates tree growth, reflected in Scan 3 branch ends extending further from the center than Scan 1 branch ends.

**Figure 8:**
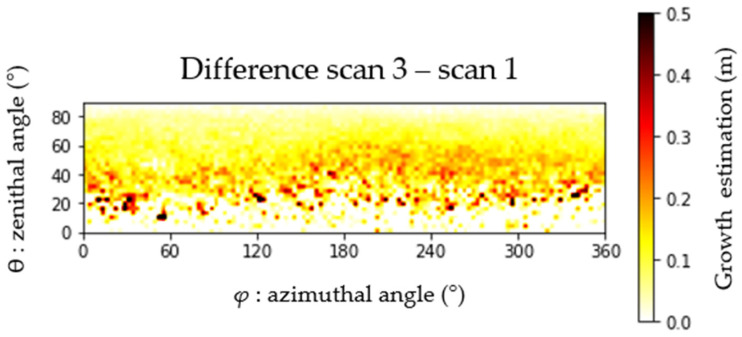
Scan 1 and Scan 3 radial distance differences represented as an image, reflecting tree growth over 37 weeks (spring to winter).

Growth was calculated for each tree as the difference between images for Scan 3 and Scan 1. The result for an individual tree, again for a resolution of 5°, is shown in Figure 8. The image, composed of 1296 cells/pixels (18 × 72), graphically represents how growth varies across the spherical coordinate system. For a growth up to 0.5 m, we can see that the higher growth rates are located below 60° (zenithal angle).

Table 2 shows the mean growth for all the trees, as well as the mean SD, calculated as the difference between the Scan 3 and Scan 1 images. Note that the values of both statistics are quite similar at different resolutions. The SD is quite high compared to the mean, due to heterogenous tree/branch growth, ranging between no growth and average growth of about 50 cm.

**Figure 9:**
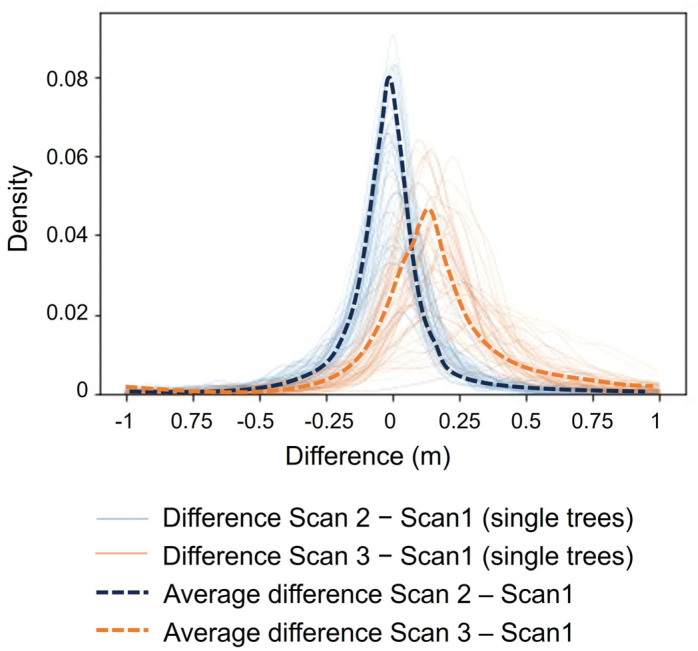
Probability density functions of the differences in each tree and the average differences between Scans 1 and 2 (blue curves) and Scans 1 and 3 (orange curves) over 37 weeks.

Figure 9 shows probability density functions for the difference in distances between Scan 2 and Scan 1 (blue curve), and between Scan 3 and Scan 1 (orange curve). The fact that the blue curve is centered around 0, as expected, while the orange curve is shifted to the right, indicates tree growth over time. The SD also increased over time, as expected, given that not all the trees have the same growth rate.

**Figure 10:**
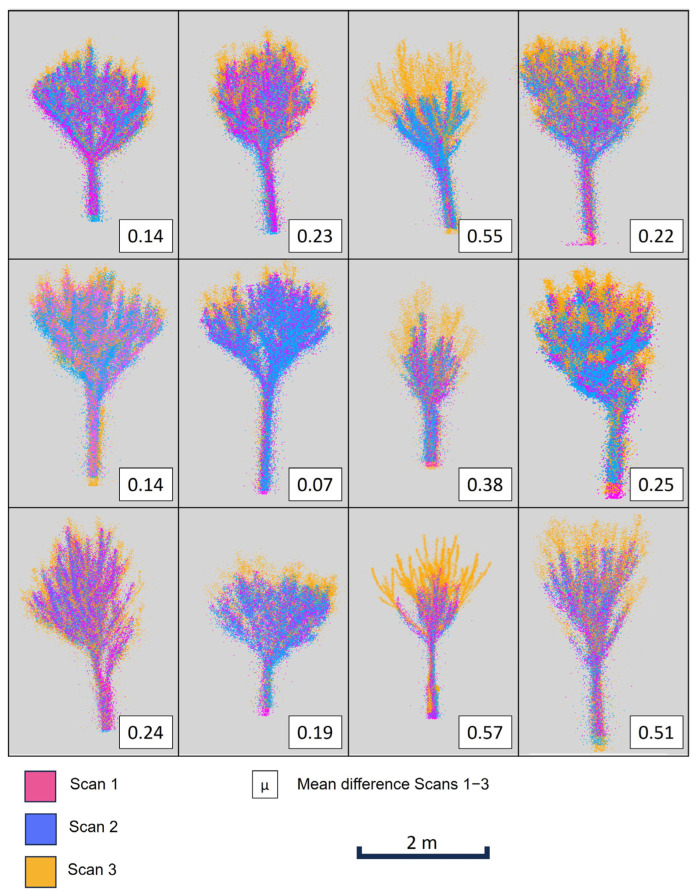
Point clouds of Scans 1, 2, and 3 (depicted using different colors) for 12 trees, plotted at the same time, reporting mean growth for each tree (i.e., the mean difference between Scans 3 and 1).

Figure 10 shows the tree scans for 12 different trees, together with the average difference calculated at a resolution of 5°. Different growth rates and how they are related with the average difference can be easily appreciated in the different trees. As mentioned, growth is not uniform, but can range from zero to 50 cm.

**Figure 11:**
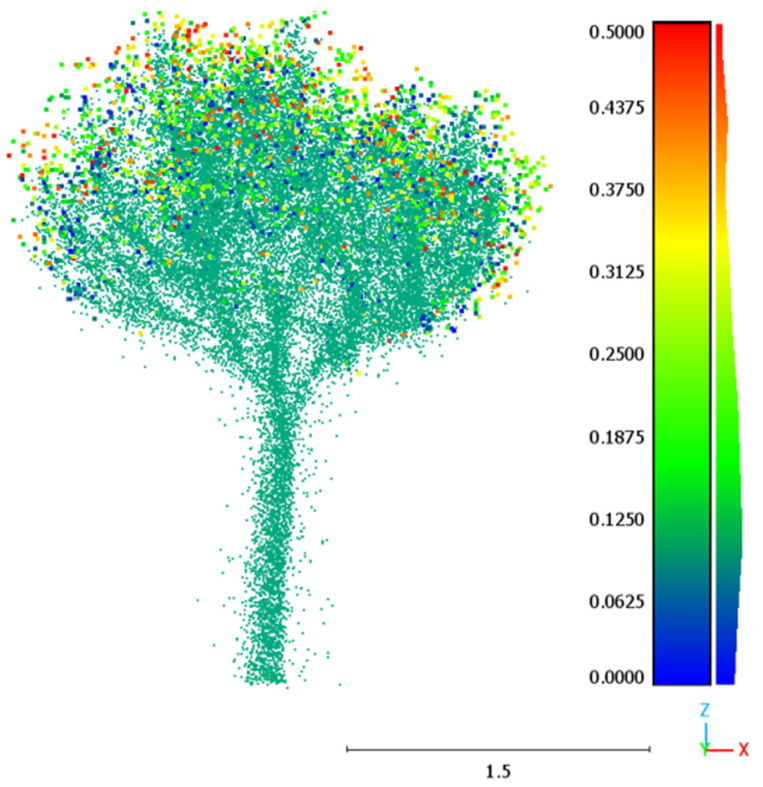
Tree growth as calculated for a reference point cloud. Scan 1, reflecting the tree at the time of the reference scan, is indicated by green points. The colored points, ranging from blue to red (0 to 0.5 m), indicate ends of branches of different lengths.

Figure 11 represents growth in a spatially explicit way, with points colored according to growth (difference between Scans 3 and 1).

## 4. Conclusions

This work describes an accurate and easily implemented non-invasive methodology for monitoring tree growth based on an HHLS technique.

The methodology was applied to 64 chestnut trees in the same orchard. Each tree was scanned three times in leaf-off conditions. Validating the methodology were two scans made almost simultaneously in time, which confirmed that both mean growth and SD were almost 0. The third scan, taken 37 weeks after the first scan, was used to estimate mean growth for both the orchard and each tree. The results obtained reflect very heterogeneous growth, ranging from zero to 50 cm.

Our results confirm that this method accurately estimates mean growth at the tree level. The method can also be extended to growth estimates of specific areas of the tree crown and even individual branches. Our results corroborate previous work that points to the usefulness of HHLS for precision agriculture and forestry purposes, not only to estimate stem and crown parameters but also to evaluate tree growth within agroforestry systems. The method could also be used to monitor and evaluate the extent to which growth is affected by infestations such as the chestnut tree wasp.

## Figures and Tables

**Table 1:** Analysis of discrepancies between Scans 1 and 2 at different resolutions (angle increments). Resolution is expressed in degrees. ME and MAE are the mean error and the mean absolute error of the distances, respectively, and σ is the standard deviation of the mean differences in distances between Scans 1 and 2.

Resolution (°)	0.5	1	2	3	5	7.5	10	15	20
ME (m)	−0.007	−0.022	−0.027	−0.020	−0.009	0.002	0.005	0.008	0.007
MAE (m)	0.026	0.033	0.034	0.029	0.022	0.021	0.022	0.023	0.022
σ (m)	0.033	0.034	0.031	0.027	0.027	0.029	0.027	0.029	0.028

**Table 2:** Mean $\left(\mu \right)$ and standard deviation $\left(\sigma \right)$ in the entire orchard obtained comparing Scan 1 and Scan 3 at different resolutions.

Resolution (°)	0.5	1	2	3	5	7.5	10	15	20
μ (m)	0.172	0.179	0.205	0.222	0.234	0.226	0.216	0.206	0.227
σ (m)	0.130	0.145	0.163	0.161	0.148	0.132	0.121	0.115	0.124

## Data Availability

Data are contained within the article.
